# Molecular Features Associated with a High-Risk Clinical Course in Neuroblastomas Initially Diagnosed as Non-High-Risk

**DOI:** 10.3390/cancers18020235

**Published:** 2026-01-12

**Authors:** Rixt S. Bruinsma, Wendy W. J. de Leng, Marta F. Fiocco, Miranda P. Dierselhuis, Karin P. Langenberg, Jan J. Molenaar, Lennart A. Kester, Max M. van Noesel, Godelieve A. M. Tytgat, Cornelis P. van de Ven, Marc H. W. A. Wijnen, Ronald R. de Krijger, Alida F. W. van der Steeg

**Affiliations:** 1 Princess Máxima Center for Pediatric Oncology, 3584 CS Utrecht, The Netherlands; 2 Department of Pathology, University Medical Center Utrecht, 3584 CX Utrecht, The Netherlands; 3 Department of Biomedical data Science, Section Medical Statistics, Leiden University Medical Center, 2333 ZC Leiden, The Netherlands; 4 Mathematical Institute, Leiden University, 2300 RA Leiden, The Netherlands; 5 Division Imaging & Cancer, University Medical Center Utrecht, 3584 CX Utrecht, The Netherlands

**Keywords:** neuroblastoma, low-risk, intermediate-risk, non-high-risk, genetics, SCA, segmental chromosomal aberrations, prognosis

## Abstract

**Background/Objectives**: Some patients initially diagnosed with non-high-risk neuroblastoma follow a high-risk clinical course and have poor survival compared to those initially diagnosed with high-risk neuroblastoma. We aimed to identify molecular aberrations present at diagnosis that may explain the high-risk clinical course in this patient group. **Methods**: Data were collected from non-high-risk neuroblastoma patients diagnosed at our center between 2014 and 2021. Segmental chromosomal aberrations (SCAs), gene amplifications and mutations at diagnosis were detected by a single-nucleotide polymorphism array and next-generation sequencing. Telomere maintenance mechanisms (TMMs) were investigated using fluorescent in situ hybridization, whole genome sequencing (WGS) and RNA sequencing. SCA counts were imputed by using multiple imputation. **Results**: The total cohort included 89 patients. Thirteen patients developed a high-risk clinical course (group A) due to progression (n = 4), local relapse (n = 4), refractory disease (n = 3) or metastases (n = 2). Seventy-six patients followed a non-high-risk clinical course (group B). An SCA profile (≥1 SCA) was present in 76% of patients in group A and only 15% in group B (*p* = 0.004). 1p deletion was associated with a high-risk clinical course (*p* = 0.034). Gains of 1q, 2p and 17q, and deletions of 4p and 11q were more common in group A. After imputation, SCA count was associated with a high-risk clinical course (pooled OR 1.256 with 95% CI 1.006–1.568, *p* = 0.044). Two patients, both group A, exhibited *MDM2*/*CDK4* amplification. Alternative lengthening of telomeres (ALT) was activated in 57% of group A. **Conclusions**: SCA profile and 1p deletion are associated with a high-risk clinical course. ALT activation, *MDM2*/*CDK4* co-amplification, SCA count, gains of 1q, 2p, and 17q, and deletions of 4p and 11q may also be relevant molecular markers. Larger studies are needed for confirmation of these findings.

## Simple Summary

Some children are initially diagnosed with a non-high-risk neuroblastoma but later develop a high-risk clinical course. It is not yet clear why this happens. We examined tumor samples taken at diagnosis to see whether the molecular profile can help to predict which children will develop a high-risk clinical course. We compared tumor samples of non-high-risk neuroblastoma patients who did develop a high-risk clinical course with those who did not. We discovered that segmental chromosomal changes, especially 1p deletion, are associated with a high-risk clinical course. Other genetic changes (activation of alternative lengthening of telomeres, *MDM2*/*CDK4* co-amplification, gains of 1q, 2p, and 17q, and deletions of 4p and 11q) might also be relevant. Larger studies are needed to confirm these findings.

## 1. Introduction

Neuroblastoma is a pediatric tumor of the sympathetic nervous system known for its clinical and molecular heterogeneity [1,2]. To optimize survival outcomes while minimizing treatment-related toxicity, treatment strategies classify patients into low-, intermediate-, or high-risk groups according to the International Neuroblastoma Risk Group (INRG) classification system [2,3]. The current INRG classification system includes age at diagnosis, disease stage, histological subgroup, tumor cell ploidy, *MYCN* status and 11q aberrations [1,3]. Treatment of non-high-risk patients (low- or intermediate-risk) varies from ‘watchful waiting’ to surgery with or without (neo)adjuvant chemotherapy [1]. This approach results in a 5-year event-free survival (EFS) of 85–90% and 5-year overall survival (OS) over 95% [4]. High-risk patients are treated with multimodal therapy, including surgery, chemotherapy, radiotherapy, high-dose chemotherapy with autologous stem cell rescue, and targeted therapy and immunotherapy, resulting in a 5-year EFS of 50% and a 5-year OS of approximately 60% [1,4]. Consequently, most neuroblastoma research focusses on the high-risk population. However, patients initially diagnosed with non-high-risk neuroblastoma who develop a high-risk clinical course due to relapse or refractory disease show even lower 5-year OS than those initially classified as high-risk [5]. This emphasizes the need for a more accurate risk stratification.

Recently, a systematic review was conducted on the prognostic value of molecular aberrations in non-high-risk neuroblastoma [6]. This review revealed a significant impact of segmental chromosomal aberrations (SCAs), specifically 1p aberrations, 2p gain and 17q gain, on prognosis. Moreover, a higher number of chromosomal breakpoints, related to the number of SCAs, is associated with poor survival rates in *MYCN*-non-amplified neuroblastoma. [7] However, research focusing on non-high-risk neuroblastoma is limited and further studies are needed to better understand the molecular profile of non-high-risk neuroblastoma patients who experience adverse outcomes. Most existing studies either target the overall neuroblastoma population or focus on high-risk neuroblastoma. Lerone et al. (2021) provided an overview of molecular aberrations associated with poor prognosis in neuroblastoma, identifying gene amplifications (*MYCN*, *ALK*, *MDM2*, *CDK4* and *FRS2*), gene mutations (*ALK*, *POHX2B*, *CHD5*, *SHANK2*, *FGFR1* and *BRAF*), *LIN28B* transcriptional activation, alternative splicing of *BARD1*, and duplication of *LMO1*, as unfavorable features [8].

Furthermore, telomere maintenance mechanisms (TMMs) are strongly associated with poor outcomes in high-risk neuroblastoma. In neuroblastoma, TMMs involve telomerase activation, through *TERT* rearrangements or *MYCN* amplification, or alternative lengthening of telomeres (ALT) (partly explained by *ATRX* alterations). Multiple studies have reported TMMs to be absent in non-high-risk tumors [8,9,10,11]. However, Roderwieser et al. (2019) showed that 10.9% (5/46) of the *TERT*-rearranged tumors were initially classified as non-high-risk [12]. For ALT-associated PML bodies (APBs), a hallmark of ALT-positive tumors, this was as high as 36.7% (18/49). Similarly, Hartlieb et al. (2021) reported that 36.4% of ALT-positive neuroblastomas, detected using C-circle assays, were classified as non-high-risk, even though their prognosis is similar to that of ALT-positive high-risk neuroblastomas [13].

Despite the extensive research into the molecular profile of high-risk neuroblastoma, the mechanisms that drive progression in non-high-risk neuroblastoma are poorly understood. We aim to identify molecular aberrations that might explain why some tumors, initially classified as non-high-risk neuroblastomas, follow a high-risk clinical course. Gaining insight into the molecular profile of this group could help to improve the current risk classification as well as the therapeutic approach, thereby improving outcomes for this specific subgroup.

## 2. Materials and Methods

### 2.1. Study Population and Clinicopathological Data

Patients diagnosed with low- or intermediate-risk neuroblastoma in the Princess Máxima Center from November 2014 till December 2021 with follow-up until May 2024 were included in the study.

All patients (or their parents) provided informed consent for the use of their data for research purposes or for participation in the iTHER study (Netherlands Trial Register, Trial NL5728; NL56826.078.16). The present study constitutes secondary use of these data and was approved by the institutional review board (PMCLAB2020.0093). Only primary biopsies or resections were included. Clinical pathological data were obtained from medical records, radiology reports and pathology reports. Data collection included age at diagnosis, sex, tumor location, tumor classification using the International Neuroblastoma Pathology Classification (INPC) [3], risk stratification according to the adapted Gesellschaft für Pädiatrische Onkologie und Hämatologie (GPOH) protocol [14,15], OS and EFS. OS and EFS were determined based on the latest available patient follow-up or imaging results from diagnosis till June 2024; patients alive at last contact were censored. An event was defined as progression, relapse, metastasis or death. Patients were categorized into two groups: group A = initially diagnosed as low- or intermediate-risk neuroblastoma but developing a high-risk clinical course and group B = diagnosed as low- or intermediate-risk neuroblastoma and following a non-high-risk clinical course. Patients were categorized into group A (1) due to metastases, progression or relapse of the tumor or (2) when high-risk therapy was required due to refractory disease under a medium-risk protocol.

### 2.2. Molecular Data

Data regarding the following molecular aberrations were gathered: SCAs, gene mutations and amplifications, *TERT* rearrangements, TERT mRNA expression levels, APBs and *ATRX* deletions.

#### 2.2.1. Segmental Chromosomal Aberrations

A single-nucleotide polymorphism (SNP) array was used to determine copy number status using the Infinium CytoSNP-850 K BeadChip (Illumina, San Diego, CA, USA) according to standard procedures. The visualization of the SNP array results and data analyses were performed using NxClinical (BioDiscovery, El Segundo, CA, USA). All chromosomes, except for chromosomes X and Y, were checked for copy number variations (CNVs) by one of the authors (R.S.B.) and independently checked by an experienced molecular biologist (W.W.J.d.L.). Chromosome arms 13p, 14p, 15p, 21p and 22p could not be analyzed since these regions do not contain unique SNPs and were therefore excluded from the SNP arrays.

A numerical chromosomal aberration (NCA) was defined as more than two (gain) or less than one (loss) copy of a full chromosome. When this concerned (part of) a chromosome arm, an SCA was present. The SCA count represents the total number of SCAs in all chromosomes. An SCA profile was present when one or more of the following SCAs were detected: 1p deletion/LOH (which corresponds to intermediate-risk classification [14]), 1q gain, 2p gain, 3p deletion, 4p deletion, 11q deletion or 17 gain. This definition is in line with the SIOPEN Low and Intermediate Risk Neuroblastoma European Study [16].

In older samples, Multiplex Ligation-dependent Probe Amplification (MLPA) was used to detect CNVs of chromosome arms 1p and 17q. MLPA analysis was performed according to the manufacturer’s instructions (P078 and P088, MRC Holland, Amsterdam, The Netherlands).

#### 2.2.2. Gene Mutations and Amplifications

Gene mutations were identified by targeted Next Generation Sequencing (tNGS). tNGS was performed as previously described using the Cancer Hotspot Panel (Thermo Fisher Scientific, Waltham, MA, USA) in combination with IonTorrent sequencing (Thermo Fisher) [17]. Variants were classified as pathogenic, likely pathogenic, variant of uncertain significance, likely benign or benign according to the American College of Medical Genetics and Genomics system for the interpretation of sequence variants [18]. Pathogenic and likely pathogenic variants were reported in this study. Furthermore, tNGS was also used to detect gene amplification, with amplification defined as >4 copies of the same gene. Any amplifications identified by tNGS were confirmed using the SNP array.

#### 2.2.3. Telomere Maintenance Mechanisms

Regarding TMM, we collected data on *TERT* rearrangements, amplifications and gene expression; APBs; and *ATRX* mutations and deletions.

*TERT* rearrangement, amplification, and *ATRX* mutations and deletions were detected using Whole Genome Sequencing (WGS) as previously described [19]. Data was generated on a NovaSeq6000 sequencer (Illumina, San Diego, CA, USA) and aligned to hg38 using bwamem2.SSNV; variants were identified using paired-normal samples and GATK 4. Likewise, SCNVs were also identified using GATK 4. *TERT* amplification was defined as the presence of >4 copies of this gene, not corrected for tumor cell percentage. For *ATRX* deletion, a −0.2 copy ratio log2 fold change was required.

When WGS data was not available, RNA sequencing was used to detect *TERT*-rearrangement and *ATRX* mutations as previously described [20]. Furthermore, TERT mRNA expression levels were determined using RNA sequencing.

Fluorescent in situ hybridization (FISH) was used to detected ALT-associated APBs as previously published [21]. A primary (PML antibody, rabbit, H-238, Santa Cruz Biotechnology, sc-621) and secondary antibody (1:2500 in 1X PBS, goat anti-rabbit Alexa Fluor 555, Invitrogen by Thermo Fisher Scientifc, Waltham, MA, USA, A27039) were used, followed by counterstaining with DAPI. When no APBs were detected, a *TERT* break-apart FISH was performed using a Streptavidin–Alexa-555 conjugate (1:500 in CAS-block, Invitrogen, S21381) and anti-digoxigenin-FITC (1:500 in CAS-block, Roche, Basel, Switzerland, 11 207 741 910) antibodies. Again, slides were counterstained using DAPI. A clinical scientist in molecular pathology examined all the slides. Both ALT-FISH and *TERT* break-apart FISH were only performed for group A patients.

### 2.3. Statistical Analysis

All patients initially diagnosed with non-high-risk neuroblastoma were categorized into two groups based on whether they developed a high-risk clinical course (group A) or followed a non-high-risk clinical course (group B). Median follow-up times were estimated with the reverse Kaplan–Meier method [22]. Furthermore, all patients were stratified based on SCA profile: SCA profile vs. no SCA profile. Fisher’s exact test was used to test the association between a high-risk clinical course and SCA profile. Kaplan and Meier’s methodology was employed to estimate OS and EFS since diagnosis. A log-rank test was used to assess the difference in survival between groups. The association between individual SCAs (deletion of 1p, 3p, 4p and 11q, and gain of 1q, 2p and 17q) and a high-risk clinical course was tested using Fisher’s exact test. Samples analyzed with MLPA were excluded from the analysis regarding the SCA profile. They were used to test the association of a high-risk clinical course with 1p deletion and 17q gain.

A univariate logistic regression model was estimated to assess the effect of SCA count on clinical course, with group B as the reference category. Complete data on CNVs were not available for older samples; therefore, missing data were imputed using fully conditional specification with the Markov Chain Monte Carlo method, allowing a maximum of 10 iterations. Predictive Mean Matching was used as the model type for continuous variables, selecting a complete case from the closest 5 predictions. A total of 20 imputed datasets were generated by assuming missing at random. The following variables were used in the imputation model: age at diagnosis, histological subgroup, high-risk clinical course (group A vs. group B), event (yes/no), survival (yes/no) and SCA count. After imputation, the same model was estimated on each imputed dataset. A pooled odds ratio (OR) according to Rubin’s Rule, along with 95% CI, was reported [23].

To compare differences in mean TERT mRNA expression levels between group A and group B, an independent-sample t-test was conducted.

SPSS for Windows, version 30.0.0.0. (IBM, Armonk, NY, USA), was used to perform statistical analysis. A *p*-value < 0.05 was considered statistically significant.

## 3. Results

A total of 89 patients with non-high-risk neuroblastoma were included in this study: 13 patients who developed a high-risk clinical course (group A) and 76 patients who followed a non-high-risk clinical course (group B). These thirteen patients were classified in group A due to progression (n = 4), relapse (n = 4), refractory disease (n = 3) or metastases (n = 2). The mean age at diagnosis was 2.15 years in group A and 3.26 years in group B. Table 1 shows further clinical characteristics of all the included patients. Table 2 provides further details regarding the group A patients. The median follow-up was 57 months in group A and 66 months in group B. 

### 3.1. Segmental Chromosomal Aberrations

An SNP array was performed in 9/13 patients in group A and 39/76 patients in group B. In group A, 6/9 patients (67%) showed an SCA profile. In contrast, 6/39 patients (15%) in group B showed an SCA profile (*p* = 0.004). All individual SCAs were relatively more common in group A, with the exception of 3p deletion. 1p deletion was the only individual SCA significantly associated with high-risk clinical course (*p* = 0.034). More details are provided in Table 3.

**Figure 1:**
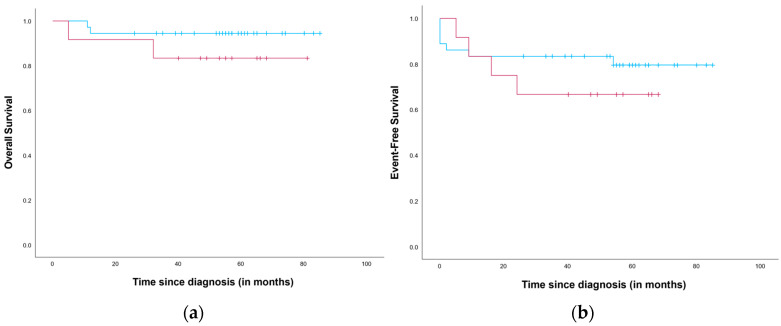
Survival after diagnosis with non-high-risk neuroblastoma. (**a**) Overall survival and (**b**) event-free survival for patients with (red) and without an SCA profile (blue).

OS and EFS from diagnosis for neuroblastomas with an SCA and without an SCA profile were estimated (Figure 1). The median follow-up was estimated at 57 months (range 5–107 months) and 56 months (range 0–103 months) for OS and EFS, respectively. The estimated 5-year OS was 83.3% (S.E. 10.8%) for patients exhibiting an SCA profile and 94.4% (S.E. 3.8%) for the remaining patients. The 5-year EFS was 66.7% (S.E. 13.6%) and 80.6% (S.E. 7.0%) for patients with and without an SCA profile, respectively. For both OS and EFS, the difference was not significant (*p* = 0.231 and *p* = 0.373, respectively).

**Table 2:** Patient characteristics and survival outcomes for group A.

Case	Age at Diagnosis	Sex	Stage	Risk Group	Mutation *	Amplification *	SCA Profile	ALT Activation **	TERT Rearrangement ***	TERT mRNA Expression (CPM)	Observed ET (m)	Type Event	Status	Observed OST (m)
1	0	F	2	LR	No	No	-	-	-		10	Progression	Alive	90
2	0	M	4S	MR	No	No	-	Yes	-		5	Progression	Deceased	23
3	0	M	4S	MR	No	No	Yes	-	No	0.0112	16	Metastases	Alive	81
4	0	M	4S	MR	No	No	Yes	No	No		68	Refractory	Alive	68
5	0	F	4S	MR	No	No	Yes	No	No		66	Refractory	Alive	66
6	5	F	2	LR	No	*MDM2* and *CDK4*	Yes	Yes	No	0	9	Relapse	Deceased	32
7	3	M	2	LR	No	*MDM2* and *CDK4*	Yes	Yes *	-		24	Relapse	Alive	53
8	0	F	4S	MR	No	No	No	-	No		0	Progression	Deceased	11
9	6	F	3	MR	*ALK*	No	-	-	No	1.3176	5	Progression	Deceased	20
10	0	M	4S	MR	No	No	Yes	No	No	0.0061	5	Refractory	Deceased	5
11	0	M	2	LR	No	No	No	Yes	No	0	2	Metastases	Deceased	12
12	8	F	2	LR	No	No	No	-	No	0.0020	54	Relapse	Alive	57
13	0	M	3	MR	No	No	-	-	-		22	Relapse	Deceased	26

* tNGS was performed in all 13 cases and WGS in 8 cases (cases 5–12). The Cancer Hotspot Panel used for tNGS did not include *ATRX* and *TERT*. Furthermore, in 3/9 cases in which WGS was performed, data on *TERT* amplifications were unavailable due to the use of an earlier version of the WGS analysis pipeline (cases 6 and 8–9). Therefore, the absence of a *TERT* amplification or a *ATRX* mutation could only be confirmed in 5 cases (cases 5, 7 and 10–12). ** The presence or absence of ALT activation was determined using ALT-FISH in 6 cases (cases 2, 4–6 and 10–11). In one case (case 7), *ATRX* deletion was detected using WGS, but no ALT-FISH was performed, so ALT activation could not be confirmed. *** Absence of *TERT* rearrangement was determined by WGS in 7 cases (cases 5–6 and 8–12), all of which were confirmed by RNA sequencing. In case 3, the absence of *TERT* rearrangement was determined by RNA sequencing alone, and in case 4, by *TERT* break-apart FISH. Abbreviations: ALT, alternative lengthening of telomeres; CPM, counts per million reads mapped; ET, event time; F, female; LR, low-risk; M, male; m, months; MR, medium-risk; OST, overall survival time; SCA, segmental chromosomal aberration; tNGS, targeted next-generation sequencing.

**Table 3:** Segmental chromosomal aberrations.

	Group A N (%)	Group B N (%)	*p*-Value
1p deletion (n = 69)			0.034
Yes	3 (25)	2 (4)	
No	9 (75)	55 (96)	
1q gain (n = 48)			0.343
Yes	1 (13)	1 (3)	
No	8 (87)	38 (97)	
2p gain (n = 48)			0.086
Yes	2 (22)	1 (3)	
No	7 (78)	38 (97)	
3p deletion (n = 48)			NA
Yes	0 (0)	1 (3)	
No	9 (100)	38 (97)	
4p deletion (n = 48)			0.343
Yes	1 (13)	1 (3)	
No	8 (87)	38 (97)	
11q deletion (n = 48)			0.159
Yes	3 (33)	5 (13)	
No	6 (67)	34 (87)	
17q gain (n = 69)			0.309
Yes	5 (42)	15 (26)	
No	7 (58)	42 (74)	
SCA profile (n = 48)			0.004
Yes	6 (67)	6 (15)	
No	3 (33)	33 (85)	

Abbreviations: NA, not applicable; SCA, segmental chromosomal aberration.

The mean SCA count was 6.11 for group A (n = 9) and 3.26 for group B (n = 39). The OR for an SCA count associated with a high-risk clinical course was 1.209 (95% CI 0.978–1.496). After imputation for the independent variable SCA count, the pooled OR was 1.256 (95% CI 1.006–1.568). An overview of SCAs identified in groups A and B is provided in Tables S1 and S2, accompanied by a legend depicted in Figure S1.

### 3.2. Gene Amplifications and Mutations

*MDM2*-*CDK4* co-amplification was detected in two patients categorized in group A, whereas these amplifications were not found in group B (Table 1 and Table 2).

One *ALK* mutation was detected in group A (Arg1275Gln) and seven *ALK* mutations, in six different patients, were detected in group B (4x Arg1275Gln, 2x Phe1174Cys and 1x Leu1196Met).

No other mutations were detected in group A, whereas group B harbored one patient with an *ERBB2* mutation and one patient with a *PTPN11* mutation.

### 3.3. Telomere Maintenance Mechanisms

In group A, WGS was performed in 8/13 cases, RNA sequencing in 8/13 cases, ALT-FISH in 6/13 cases, and *TERT*-break apart was utilized in 3/13 cases. In 3/8 cases where WGS was performed, data on *TERT* amplifications and *ATRX* deletions were unavailable due to the use of an earlier version of the WGS analysis pipeline. No *TERT* rearrangements or *TERT* amplifications were identified. However, three samples were positive for APBs and one other sample showed an *ATRX* deletion. ALT-FISH was not performed on the latter sample, precluding confirmation of ALT activation.

In group B, WGS was conducted in 15/76 cases and RNA sequencing in 18/76 cases. Data regarding *TERT* amplifications and *ATRX* deletion were unavailable for one case in which WGS was performed. No *TERT* rearrangements, *TERT* amplifications, *ATRX* mutations or *ATRX* deletions were detected.

TERT mRNA expression levels were available in 6/13 cases in group A and 18/76 cases in group B. The median mRNA expression level was 0.0041 CPM in group A and 0.0091 CPM in group B (*p* = 0.381). One outlier was observed in group A, with an mRNA expression level of 1.32 CPM. An earlier version of the WGS analysis pipeline was used for this sample, resulting in the absence of data on *TERT* amplification.

## 4. Discussion

This study investigated molecular aberrations associated with a high-risk clinical course in patients initially diagnosed with non-high-risk neuroblastoma. We have investigated the SCA profile, individual SCAs, gene mutations and amplifications, and the activation of TMMs by either *TERT* rearrangements or ALT.

There was a significant association between SCA profile and a high-risk clinical course. OS and EFS were also worse in patients with an SCA profile, although the difference was not statistically significant. Furthermore, gains of 1q, 2p and 17q, as well as deletions of 1p, 4p and 11q, were more common in patients who developed a high-risk clinical course. This difference was statistically significant for 1p deletion. When focusing on SCA count, there was no statistically significant association between SCA count and high-risk clinical course. After imputation, the OR remained similar, but the association reached statistical significance. Research into the robustness of multiple imputation using chained equations has shown high robustness for missing values up to 50% [24]. In our study, the proportion of missing data was 46% (41/89). As the majority of missing data concerns older samples obtained prior to the implementation of SNP array analysis at our center, the availability of molecular data is unlikely to be associated with specific clinical or biological characteristics within this group. Identifying the SCA profile, SCA count and individual SCAs (specifically 1q, 2p and 17q gains, and 1p, 4p and 11q deletion) as unfavorable features for non-high-risk neuroblastoma would also be in line with previous research [6,7,25].

*MDM2*-*CDK4* co-amplification, located at 12q13.3-q14.1 (CDK4) and 12q15-q21.1 (MDM2) [26,27], was observed in two neuroblastomas in our cohort, both of which developed a high-risk clinical course. Prior research also suggests an association between *MDM2*-*CDK4* co-amplification and poor prognosis [26,27,28,29]. Amoroso et al. (2020) compared the survival of eight non-high-risk neuroblastomas harboring 12q co-amplifications (*MDM2*/*CDK4*/*FRS2*) with 170 non-high-risk neuroblastomas without such amplifications, and found poor outcomes in the former group [27]. Similarly, Martinez-Monleon et al. (2022) reported extremely poor survival in nineteen 12q-amplified neuroblastomas, even in comparison to *MYCN*-amplified neuroblastomas [26]. Eighteen of these neuroblastomas exhibited *MDMD2* and/or *CDK4* amplification. Inomistova et al. (2025) found significantly better EFS in patients without *MDM2* amplification, and Gundem et al. (2024) showed an association between *MDM2*-*CDK4* amplification and poor outcomes [28,29]. The latter study suggested that, in some patients, co-occurring *ATRX* alterations and *TERT* overexpression may have contributed to these dismal outcomes. However, Gundem et al. investigated high-risk neuroblastomas, whereas our study focuses on non-high-risk neuroblastomas, in which TMM seem to be absent [8,9,10,11]. The prognostic impact of *MDM2*-*CDK4* co-amplification might be explained by the regulating role of *CDK4* in cell cycle progression and the autoregulatory feedback loop by which *MDM2* suppresses p53 [26,30,31,32,33].

*ALK* mutations are known to be associated with poor outcomes in high-risk neuroblastoma patients [34,35,36]. However, in our study, the proportion of tumors harboring an *ALK* mutation was similar between neuroblastomas following a high-risk and non-high-risk clinical course. This finding is most likely due to our study population comprising solely non-high-risk neuroblastomas. Previous studies have similarly shown no significant difference in survival between *ALK*-mutated versus *ALK*-WT neuroblastomas within non-high-risk, low-risk or *MYCN*-non-amplified subgroups [34,35,37].

Previous studies have shown that both telomerase and ALT activation can occur in non-high-risk neuroblastomas and that the presence of TMMs is associated with worse prognosis [12,13]. In the present study, no *TERT* rearrangements were detected. This is likely due to the low prevalence of this aberration in non-high-risk neuroblastomas. Roderwieser et al. (2019) identified *TERT* rearrangements in 5/236 (2.1% or 1:47 patients) non-high-risk neuroblastomas [12]. Therefore, the absence of *TERT* rearrangements in our study likely reflects the small number of WGS-analyzed cases (n = 27). Notably, one non-high-risk neuroblastoma that developed a high-risk clinical course showed elevated TERT mRNA expression levels (Figure 1, Table 2). The upregulation of TERT, consequently inducing telomerase activity, may have been caused by *TERT* amplification or a *TERT* promotor mutation [38,39]. Unfortunately, data on these specific aberrations were not available for this patient. Nevertheless, the elevated TERT expression likely contributed to disease progression, and therefore upstaging, of this tumor [9,11,39]. Due to limited availability of RNA sequencing data in our cohort, this hypothesis could not be substantiated.

ALT activation was confirmed in 3/6 cases (50%) in which ALT-FISH was performed, and an *ATRX* deletion was detected in an additional patient. All four patients developed a high-risk clinical course. Unfortunately, no ALT-FISH was performed in patients who followed a non-high-risk clinical course. However, the percentage of ALT activation observed in patients who developed a high-risk clinical course was substantially higher than what has generally been reported in non-high-risk neuroblastomas. Hartlieb et al. (2021) detected ALT activation in 24/449 non-high-risk neuroblastomas (5.3%) and Roderwieser et al. (2019) in 18/142 cases (12.7%) [12,13]. This suggests that ALT activation may be associated with a high-risk clinical course. However, no firm conclusions can be drawn since ALT-FISH was not utilized in patients following a non-high-risk clinical course.

This study focused on patients initially diagnosed with non-high-risk neuroblastoma who developed a high-risk clinical course. Despite the extremely poor survival of this specific subgroup, it has received little attention in previous research, underscoring the relevance and value of our research [5]. However, the rarity of both this subgroup and the molecular aberrations examined in combination with missing data on TMM compromised our ability to reach robust conclusions. In the case of *MDM2*-*CDK4* co-amplification, the sample size was too small to perform statistical analyses. Another limitation is the absence of ALT-FISH data and scarcity of WGS data in patients who followed a non-high-risk clinical course, which precluded the investigation of TMMs in this subgroup.

## 5. Conclusions

Both SCA profile and 1p deletion are associated with a high-risk clinical course in patients initially diagnosed with non-high-risk neuroblastoma. Furthermore, our study suggests an association of SCA count, other individual SCAs (1q, 2p and 17q gain, and 4p and 11q deletion) and *MDM2*-*CDK4* co-amplification with a high-risk clinical course. Given the rarity of both this subgroup of patients and the molecular aberrations examined, a large multicenter study is needed to include enough patients to conduct robust statistical analyses. Should our findings be confirmed, incorporation of these molecular aberrations into the INRG classification system and other risk stratification systems should be considered. Improving current classification systems, thereby altering therapeutic approaches, may improve the survival of these seemingly non-high-risk neuroblastoma patients.

## Figures and Tables

**Table 1:** Patient characteristics.

	Group A (n = 13) (%)	Group B (n = 76) (%)
Gender		
Female	6 (46)	40 (53)
Male	7 (54)	36 (47)
Age group		
Infant (<1 year)	8 (62)	32 (42)
Child (1–18 years)	5 (38)	44 (58)
Primary tumor location		
Adrenal gland	6 (46)	29 (38)
Paravertebral ganglia	3 (23)	39 (51)
Other/unknown	4 (31)	8 (11)
Survival		
No	6 (46)	0 (0)
Yes	7 (54)	76 (100)
Event		
Yes	11 (85)	9 (12)
No	2 (15)	66 (88)
SCA profile		
Yes	6 (67)	6 (15)
No	3 (33)	33 (85)
*Missing*	*4*	*37*
*MDM2-CDK4* co-amplification		
Yes	2 (17)	0 (0)
No	10 (83)	57 (100)
*Missing*	*1*	*19*
*ALK* mutation		
Yes	1 (8)	6 (11)
No	11 (92)	51 (89)
*Missing*	*1*	*19*
ALT activation		
Yes	4 * (57)	-
No	3 (43)	-
*Missing*	*6*	*76*
TERT-rearrangement		
Yes	0 (0)	0 (0)
No	9 ** (100)	18 (100)
*Missing*	*4*	*58*

* ALT activation was detected by FISH in three patients. One patient showed an *ATRX* deletion, but no ALT-FISH was performed, so ALT activation could not be confirmed. ** Absence of *TERT* rearrangement was determined by WGS in seven cases, all of which were confirmed by RNA sequencing. In one additional case, RNA sequencing alone was used, and in another, only TERT break-apart FISH was performed. Abbreviations: ALT, alternative lengthening of telomeres; FISH, fluorescent in situ hybridization; SCA, segmental chromosomal aberration; WGS, whole genome sequencing.

## Data Availability

The datasets presented in this article are not readily available because the patients included in this research are still participating in ongoing trials. Requests to access the datasets should be directed to BDACuitgifte@prinsesmaximacentrum.nl.

## Supplementary Materials

The following supporting information can be downloaded at: https://www.mdpi.com/article/10.3390/cancers18020235/s1. Figure S1: Legend; Tables S1 and S2; Table S1: Segmental Chromosomal Aberrations Group A; Table S2: Segmental Chromosomal Aberrations Group B.

## Abbreviations

The following abbreviations are used in this manuscript:

ALT	Alternative lengthening of telomeres
APBs	ALT-associated PML bodies
CI	Confidence interval
CNVs	Copy number variations
EFS	Event-free survival
FISH	Fluorescent in situ hybridization
GPOH	Gesellschaft für Pädiatrische Onkologie und Hämatologie
INPC	International Neuroblastoma Pathology Classification
INRG	International Neuroblastoma Risk Group
MLPA	Multiplex Ligation-dependent Probe Amplification
NCA	Numerical chromosomal aberration
OR	Odds ratio
OS	Overall survival
SCA	Segmental chromosomal aberration
S.E.	Standard error
SNP	Single-nucleotide polymorphism
TMM	Telomere maintenance mechanism
tNGS	Targeted next-generation sequencing
WGS	Whole genome sequencing
