# Supplementary material for: Molecular Features Associated with a High-Risk Clinical Course in Neuroblastomas Initially Diagnosed as Non-High-Risk
Source: Cancers (Basel). 2026 Jan 12;18(2):235. doi: 10.3390/cancers18020235 (PMC12838732; doi:10.3390/cancers18020235)
Supplement: Supplementary file 1 [file cancers-18-00235-s001.zip › Figure S1.pdf]

	**No numerical chromosomal aberration**
	**Loss**
	**Gain**
	**Allelic imbalance / Loss of heterozygosity (LOH)**
	**Partial loss**
	**Partial gain**
	**Partial loss & Partial gain**
	**Partial loss & Partial allelic imbalance / LOH**
	**Partial gain & Partial allelic imbalance / LOH**
	**No molecular diagnostics performed**

Figure S1. Legend table S1 and S2.
