# Supplementary material for: Molecular Features Associated with a High-Risk Clinical Course in Neuroblastomas Initially Diagnosed as Non-High-Risk
Source: Cancers (Basel). 2026 Jan 12;18(2):235. doi: 10.3390/cancers18020235 (PMC12838732; doi:10.3390/cancers18020235)
Supplement: Supplementary file 1 [file cancers-18-00235-s001.zip › Table S1.pdf]

Table S1. Segmental Chromosomal Aberrations Group A.

	1		2		3		4		5		6		7		8		9		10		11		12		13		14		15		16		17		18		19		20		21		22	
	p	q	p	q	p	q	p	q	p	q	p	q	p	q	p	q	p	q	p	q	p	q	p	q	p	q	p	q	p	q	p	q	p	q	p	q	p	q	p	q	p	q	p	q
1																																												
2																																												
3																																												
4																																												
5																																												
6																																												
7																																												
8																																												
9																																												
10																																												
11																																												
12																																												
13																																												
