# Supplementary figures and images for: Molecular Features Associated with a High-Risk Clinical Course in Neuroblastomas Initially Diagnosed as Non-High-Risk
Source: Cancers (Basel). 2026 Jan 12;18(2):235. doi: 10.3390/cancers18020235 (PMC12838732; doi:10.3390/cancers18020235)

**Table S2.** Segmental Chromosomal Aberrations Group B.

[illegible]

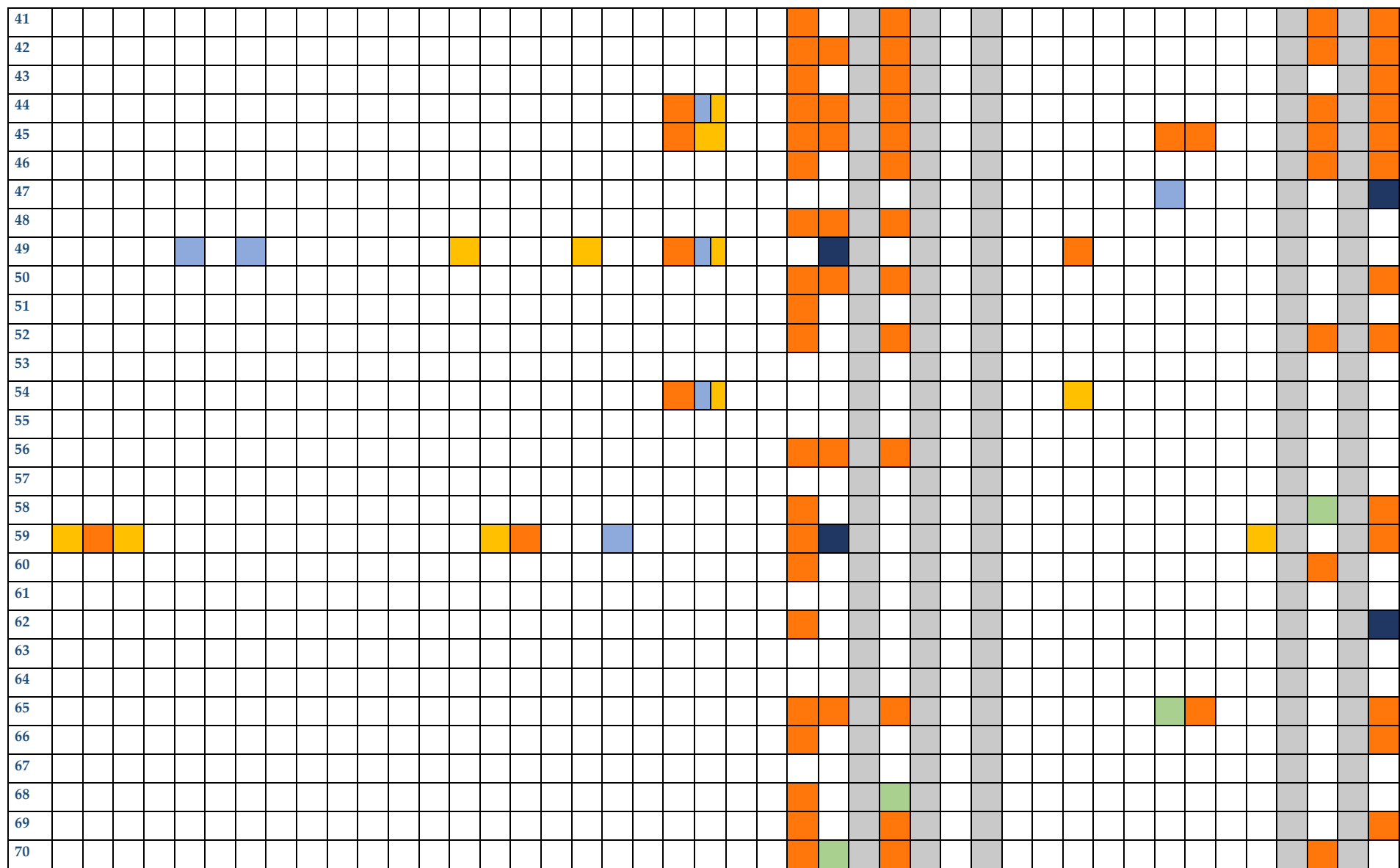

Supplement: Supplementary file 1 [file cancers-18-00235-s001.zip › Table S2.pdf]
